# Rare localized extralobar sequestration with congenital cystic adenomatoid malformation: a case report

**DOI:** 10.1186/s40792-017-0321-3

**Published:** 2017-03-21

**Authors:** Satoshi Nagasaka, Satsuki Kina, Yoshihito Arimoto, Fumi Yokote, Tsuyoshi Uchida, Hirochika Matsubara

**Affiliations:** 1 General Thoracic Surgery, National Center for Global Health and Medicine, 1-21-1 Toyama, Shinjuku-ku, Tokyo, 162-8655 Japan; 2 Second Department of Surgery, University of Yamanashi Hospital, 1110, Shimokato, Chuo-shi, Yamanashi, 409-3898 Japan

**Keywords:** Congenital cystic adenomatoid malformation, Extralobar pulmonary sequestration, Right mediastinal mass, Mediastinal extralobar sequestration

## Abstract

Extralobar sequestrations constitute a rare form of congenital pulmonary airway malformations that are difficult to diagnose. Here, we report a rare case of a localized extralobar sequestration in the right superior portion of the mediastinum accompanied by congenital cystic adenomatoid malformation.

A 19-year-old man presented with a right upper mediastinal mass that was detected using chest radiography, had a history of left spontaneous pneumothorax, and had undergone a bullectomy 4 years previously.

The initial diagnosis included a mature teratoma and a bronchogenic cyst in the mediastinum; however, the presence of a cystic mass in the right upper lobe of the lung prompted further examination. A preoperative diagnosis of extralobular sequestration was finally determined using contrast-enhanced computed tomography. The aberrant artery was connected to the brachiocephalic artery, and its drainage vein was connected to the right pulmonary artery, uniquely behind the pericardium. Despite the unique location, right mediastinal extralobular sequestration with a congenital cystic adenomatoid malformation in the right upper lobe was confirmed pathologically. Examination of contrast-enhanced chest computed tomography (CT) and three-dimensional computed tomography images enabled a correct diagnosis. It is very important for surgeons to correctly diagnose and identify an aberrant artery and drainage vein to prevent uncontrolled hemorrhage.

## Background

Pulmonary sequestrations in adults are a rare congenital anomaly. An extralobar sequestration (ELS) is the least common of all congenital pulmonary airway malformations. It is often located in the left lower lobe and diaphragm and receives its blood supply from systemic arteries, whereas its venous drainage occurs via a systemic vein. The atypical location makes it difficult to diagnose this condition correctly. Here, we report a unique case of right mediastinal ELS that was diagnosed preoperatively and successfully treated surgically.

## Case presentation

**Fig. 1:**
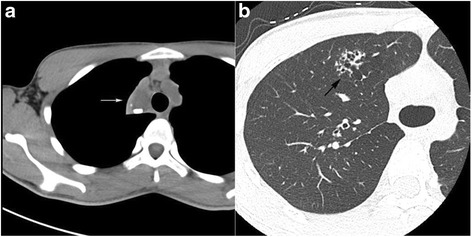
**a** Plain chest computed tomography image (axial) shows a right anterior mediastinal cystic mass with calcification (*arrow*). **b** Chest computed tomography image shows a cystic lesion in the right anterior segment (*arrow*)

A 19-year-old man was referred to our hospital for a right upper mediastinal mass that was detected on chest radiographic examination. The patient had no symptoms; however, he had a medical history of left spontaneous pneumothorax and had undergone a bullectomy 4 years previously. Plain chest computed tomography (CT) revealed a multilobulated cystic mass that was sharply defined and triangular shaped, measuring 52 × 31 × 25 mm^3^ with calcification, and surrounded by the right innominate vein, the right upper lobe of the lung, and the trachea (Fig. 1a). Initial diagnoses revealed a mature teratoma and a bronchogenic cyst. On the other hand, localized multicystic lesions were detected in the right anterior segment of the lung, which were completely separated from the mediastinal tumor (Fig. 1b). Despite the patient’s medical history, considering his age, the lesion on the inner side of the lung suggested not only the impossibility of a pneumothorax but also other potential underlying diseases.

Contrast-enhanced chest CT revealed strong enhancement around the tumor and an aberrant artery originating from the brachiocephalic artery and draining into the right main pulmonary artery behind the pericardium. Despite the unique location, ELS with a congenital cystic adenomatoid malformation (CCAM) of the right upper lobe was strongly suspected.

**Fig. 2:**
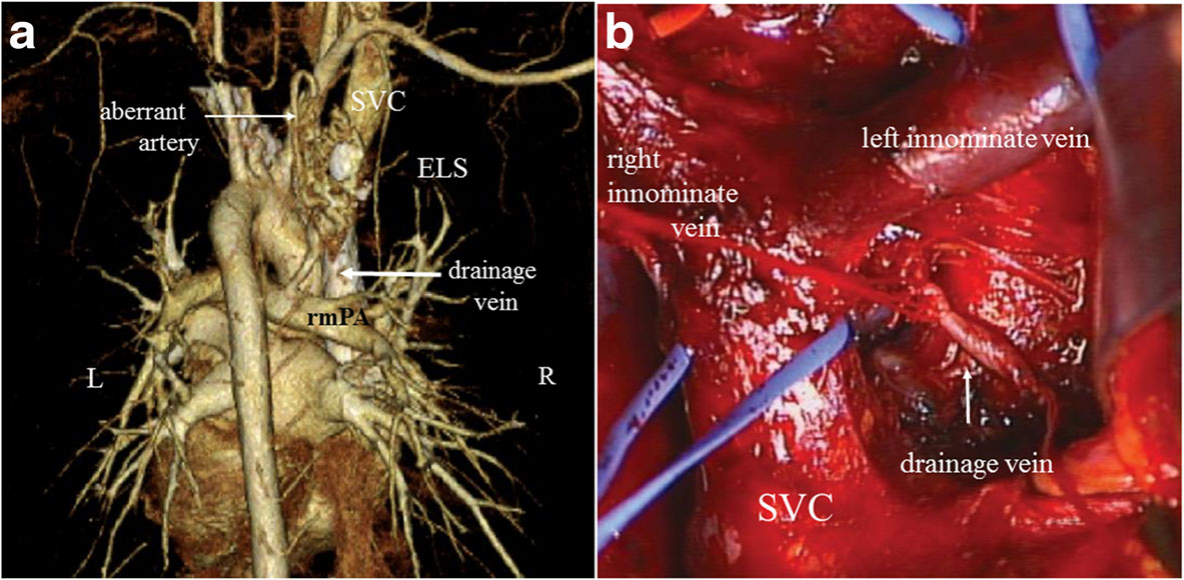
**a** Three-dimensional reconstruction computed tomography image (posterior view) shows an aberrant artery branching out from the brachiocephalic trunk and the vein draining into the right pulmonary artery. **b** Intraoperative findings show the drainage vein (ligated; *arrow*) to the *right* pulmonary artery behind the dorsal pericardium (cut away) between the ascending aorta (pulled to the *left side*) and the superior vena cava(SVC). The *right* and *left* innominate veins and the SVC are taped. *ELS* extralobar sequestration, *rmPA* right main pulmonary artery, *SVC* superior vena cava

The tumor was examined in detail using three-dimensional CT (Fig. 2a). No other regions with associated abnormalities were detected on further examination, except for the cystic lesion in the right anterior segment.

A sternotomy was performed, and a soft heterogeneous mass with its own visceral pleura was detected surrounding the right and left innominate veins. Arteries originating from the brachiocephalic artery were identified, which were also visible on three-dimensional CT. The drainage vein was followed in the pericardium and ligated at the top of the right main pulmonary artery behind the dorsal pericardium (Fig. 2b). The mediastinal mass shrank after ligating the aberrant artery and the drainage vein. The cystic mediastinal tumor, which existed independently in the mediastinal pleura, was completely removed. In addition, the cystic mass in the right upper lobe was partially resected.

**Fig. 3:**
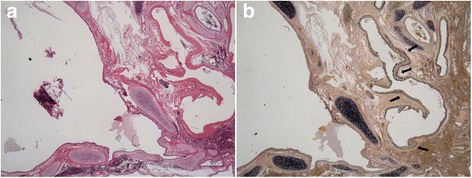
**a** ELS. Histological findings reveal that the dilated cyst wall is covered with ciliated columnar epithelium, surrounded by cartilage and bronchial glands (hematoxylin and eosin staining, original magnification ×10). **b** Elastica van Gieson staining showed elastic fibers in the intimal layer of the tortuous artery (*arrow*) (original magnification ×10)

**Fig. 4:**
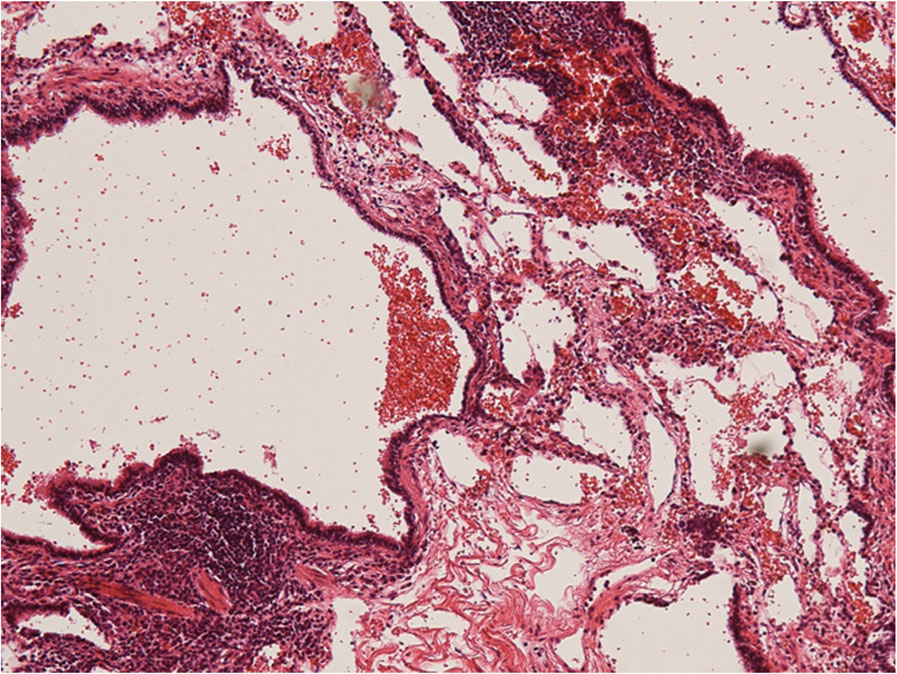
CCAM. The surgical specimen of the lung shows that the wall of the cyst is lined with cuboidal and pseudostratified, respiratory-like epithelium with chronic inflammatory cell infiltration. (hematoxylin and eosin staining, original magnification ×40)

The macroscopic findings indicated a mediastinal mass that measured 48 × 28 × 23 mm^3^ and was encased within its own pleura. Histological findings revealed that the cyst wall was covered with ciliated columnar epithelium and surrounded by cartilage and bronchial glands. Hematoxylin and eosin staining showed that the tortuous artery was thick walled (Fig. 3a), and Elastica van Gieson staining identified elastic fibers in the intimal layer of the tortuous artery (Fig. 3b). These findings were compatible with ELS. The surgical specimen of the right anterior segment of the lung revealed that the wall of the cyst was lined by cuboidal and pseudostratified, respiratory-like epithelium (via hematoxylin and eosin staining), which led to the diagnosis of CCAM (Fig. 4). There was no evidence of malignancy in the ELS or CCAM.

In addition, the specimen of his previous pneumothorax showed normal bulla and blebs of the lung.

The patient’s postoperative course was excellent. At the time of this report, he had been faring well with no postsurgical complications for 2 years.

## Conclusions

In adult patients, pulmonary sequestrations are a rare congenital malformation of the lung. However, they are the second most common congenital lung anomaly in pediatric respiratory disease because they are typically diagnosed during childhood.

A pulmonary sequestration is a lung anomaly lacking normal tracheobronchial connections and supplied systemic arteries. Pulmonary sequestrations are divided into two types, intralobar sequestration (ILS) and ELS, based on whether the visceral pleura does or does not separate the normal lobe, respectively. An ELS is rare; they usually occur between the lower lobe of the lung and diaphragm and manifest with multiple anomalies. CCAM is also a rare developmental lung anomaly in adolescence and adulthood, but it is the most common congenital lung lesion in childhood examined by routine ultrasound. Stocker divided CCAM that are hamartomatous lesions comprising cystic and adenomatous elements into three major types based upon the size of the cysts and their cellular characteristics [1]. The present case involved type II CCAM comprising multiple cysts approximately 0.5 cm in diameter and solid areas that blended into adjacent normal tissue.

There are two major theories surrounding the source of lung anomalies: (1) they originate in the primitive foregut or lung bud and (2) they arise from the six pairs of aortic arches or venous radicals and their derivatives. Richard et al. [2] reported that proportion of ELS with frequently associated congenital cystic adenomatoid malformation type II was approximately 50%. Recently, the pathogenesis of CCAM, LLS, ELS, and lobar emphysema has been thought to have common origins in different stages of lung development and at different sites and times, under the influence of airway obstruction [3, 4]. We considered co-existence of these two diseases in the present case seems not to be coincidental. Congenital cystic adenomatoid malformation was considered a premalignant lesion, and right upper lobectomy was preferable. Therefore, careful long-term follow-up was required.

Among 133 cases of ELS reported by Savic et al. [5], only two were located in the right mediastinum. An ELS of the right superior portion of the mediastinum may be rarer. The arterial supply is normally provided by branches of the aorta, and the venous drainage is normally routed through the systemic veins, usually the inferior vena cava or azygos-hemiazygos systems. However, in this patient, the venous return was unique. The drainage vein returned to the right main pulmonary artery behind the dorsal pericardium.

Savic et al. [5] reported that only six of 66 patients who underwent surgery for ELS had received a correct diagnosis. A unique location can make a preoperative diagnosis even more difficult. However, carefully interpreting contrast-enhanced chest CT and three-dimensional CT images can ensure a correct diagnosis. An accurate diagnosis results in a safe and easy operation. Physicians should take great care in diagnosing a mediastinal tumor, regardless of whether it is in an atypical location [6–8].

Savic et al. [5] reported five patients with an ILS who died because of uncontrolled bleeding of the aberrant artery. In patients with ELS, massive intraoperative bleeding due to injured aberrant vessels has been reported [9]. It is therefore very important to identify aberrant vessels to prevent an uncontrolled hemorrhage. It is very useful to examine contrast-enhanced chest CT images and to create a reconstructed three-dimensional CT image for safe surgery.

Pulmonary sequestration with CCAM has recently been reported more frequently in pediatric surgery [2–4]. Due to the presence of a mediastinal tumor with a cystic mass in the lung, a sequestration was also suspected in this patient.

## Abbreviations

CCAM: Congenital cystic adenomatoid malformation
CT: Computed tomography
ELS: Extralobar sequestration
